# Hybrid Robust Beamforming Optimization for LEO Satellite Communications Under DOA Estimation Errors in Spectrum Sharing Scenarios

**DOI:** 10.3390/s26113501

**Published:** 2026-06-02

**Authors:** Yunfeng Wang, Xuxu Xie, Jiyang Jia

**Affiliations:** 1 College of Information Science and Engineering, Zaozhuang University, Zaozhuang 277100, China; jiajiyang@uzz.edu.cn; 2 Xinjiang Key Laboratory of Multimodal Intelligent Computing and Large Models, Kashi University, Kashi 844000, China; 3 School of Communication and Information Engineering, Nanjing University of Posts and Telecommunications, Nanjing 210003, China; 2021010109@njupt.edu.cn

**Keywords:** LEO satellite communications, spectrum sharing, robust beamforming, direction-of-arrival (DOA) estimation error

## Abstract

Low Earth orbit (LEO) satellite systems provide ubiquitous global connectivity for massive grant-free random access Internet of Things (IoT) applications. Full frequency reuse (FFR) improves spectrum efficiency in spectrum sharing scenarios but introduces severe adjacent beam and cross-system co-channel interference. Meanwhile, the high mobility of LEO satellites hinders accurate instantaneous channel state information (iCSI) acquisition, and random direction-of-arrival (DOA) estimation errors cause statistical CSI (sCSI) mismatch, which degrades beamforming performance and makes it difficult to balance transmission robustness, user fairness, and onboard computational complexity. To address these issues, we propose a low-complexity Hybrid Optimized Robust Beamforming (HORBA) algorithm. We first construct a robust joint optimization model to characterize the coupling effects of DOA errors, outdated CSI, and multi-dimensional interference, with constraints on per-user minimum SINR and cross-system interference temperature. Then, based on the block coordinate descent framework, we decouple the original non-convex problem into two convex subproblems, which are solved via generalized eigenvalue decomposition and first-order Taylor expansion, combined with an adaptive sampling mechanism that balances accuracy and complexity. Simulation results verify that our algorithm outperforms typical benchmarks in sum rate and robustness, maintains low onboard processing complexity, and effectively alleviates edge user rate polarization.

## 1. Introduction

To support the 6G vision of global ubiquitous connectivity, low Earth orbit (LEO) satellite constellations have become the core pillar of non-terrestrial networks (NTNs). These systems break the coverage limitations of terrestrial cellular networks and deliver stable services for ocean navigation, remote area access, emergency rescue and massive Internet of Things (IoT) applications [1,2,3]. With the large-scale deployment of commercial constellations like Starlink and OneWeb, the exponential growth of access demand has made spectrum resource scarcity the core bottleneck restricting LEO system performance. Spectrum sharing between co-frequency satellite systems and between satellite and terrestrial networks has thus become an inevitable trend to maximize spectrum utilization [4,5,6].

For efficient spectrum sharing, frequency reuse is the core technical means in satellite communication systems. Traditional satellite systems widely adopt the 7-color frequency reuse (FR7) scheme. This scheme suppresses inter-beam interference by allocating orthogonal spectrum resources to adjacent beams but fundamentally sacrifices spectral efficiency. Its available bandwidth for a single beam is only 1/7 of the full spectrum, which cannot meet the high-throughput demand of future IoT networks [7]. To address this limitation, full frequency reuse (FFR) has become the mainstream development direction of current spectrum sharing technology [8]. FFR enables all beams in the system to reuse the complete spectrum bandwidth, and theoretically multiplies system throughput under the same spectrum resources. To mitigate the inherent inter-beam interference of FFR, multi-beam joint processing technology has emerged as the core enabling technology. Through onboard large-scale phased array antennas, high-gain directional spot beams are generated via beamforming design in multi-beam satellite systems. These beams can compensate for the large path loss of satellite-ground links, and realize flexible interference suppression in the spatial domain to support aggressive FFR deployment in spectrum sharing scenarios [9].

However, beamforming design in multi-beam satellite systems faces unique practical challenges in LEO scenarios. Traditional algorithms developed for terrestrial networks cannot be directly adapted to the inherent characteristics of satellite-ground links. The first challenge lies in the difficulty of accurate channel state information (CSI) acquisition. The high-speed motion of LEO satellites at 7.5∼7.9 km/s relative to the ground causes severe Doppler shift and shortened channel coherence time [10,11,12]. The long round trip propagation delay of satellite-ground links further leads to serious CSI outdatedness [13,14,15].

For grant-free random access (GF-RA) scenarios widely used in satellite IoT services, statistical CSI (sCSI) is widely used to avoid real-time instantaneous CSI (iCSI) acquisition. The accuracy of these statistical channel models heavily depends on direction-of-arrival (DOA) estimation of user signals [16,17]. Fast and accurate DOA estimation must be completed within the 1∼5 ms channel coherence time of LEO satellites, which imposes stringent requirements on the computational complexity of the beamforming algorithm.

Random DOA estimation errors originate not only from atmospheric turbulence, ionospheric scintillation and terminal positioning deviation but also from the coupling with LEO satellite positioning accuracy. Meter-level onboard positioning errors will be directly converted into angular prior errors, leading to beam pointing mismatch [18]. More importantly, these DOA estimation errors not only impair communication beamforming performance but also significantly degrade the positioning accuracy of LEO satellite communication and navigation integration systems. By simple geometric calculation, even a 1° DOA estimation error will lead to approximately 19.2 km of ground positioning error at the 1100 km orbital altitude adopted in this work. As emphasized in recent studies on LEO-based coarse positioning systems [19], high-precision real-time AoA estimation is a core enabling technology for LEO navigation services, and even tenths of a degree of angular error can render the positioning results unusable for most location-based applications. This clearly defines the accuracy requirement for DOA estimation in practical LEO systems, and further highlights the necessity of our robust beamforming design, which can maintain satisfactory communication performance even in the presence of such inevitable DOA estimation errors.

These negative effects are more prominent in FFR-based spectrum sharing scenarios [20,21]. Moreover, co-frequency coexistence between heterogeneous systems introduces cross-system coupled interference, while onboard payload hardware constraints strictly limit algorithm complexity; in addition, the inherent link budget difference between beam-center and edge users leads to severe rate polarization, all of which put forward higher requirements for robust beamforming design in practical LEO deployments [22,23,24].

Extensive studies have been carried out to address the above challenges. First, we briefly clarify the relationship between DOA estimation techniques and beamforming robustness: mainstream DOA methods include subspace-based approaches (e.g., MUSIC and ESPRIT) with high accuracy but high complexity, maximum likelihood methods with good robustness but heavy computational burden, and deep learning-based methods suitable for complex scenarios but relying on sufficient training datasets. This paper does not focus on optimizing DOA estimation accuracy but investigates robust beamforming optimization under weak prior conditions of random estimation errors, which forms a complementary research direction to existing DOA estimation schemes.

For interference management in spectrum sharing scenarios, existing works mainly focus on frequency reuse optimization and user clustering strategies. Most of these works still rely on multi-color frequency reuse schemes and cannot fully exploit the spectrum efficiency gain brought by FFR [8,22]. For multi-beam transmission based on FFR, sCSI-assisted precoding schemes have laid a theoretical foundation for onboard processing without iCSI. These schemes lack consideration of DOA estimation errors and CSI uncertainty, and will suffer severe performance degradation in non-ideal scenarios [25]. For CSI mismatch problems, worst case and stochastic robust optimization methods have been proposed in existing works. Most of these methods only model a single error source without considering the coupling effect of DOA errors, outdated CSI and multi-dimensional interference [26]. They also often suffer from either over conservative design or excessive computational complexity. Existing low-complexity optimization frameworks based on block coordinate descent (BCD) also lack sufficient robustness to DOA estimation errors in spectrum sharing scenarios [27,28].

While these research efforts have made valuable progress, there are still several areas that can be further explored to better address the practical challenges of beamforming design in LEO satellite systems. First, existing works lack a complete technical logic connecting spectrum sharing mechanism evolution with beamforming design, and have not fully revealed the internal relationship between frequency reuse strategy selection and beamforming technology design in multi-beam satellite systems [18,29]. Second, existing robust models may not fully characterize the coupling effect of multiple non-ideal factors in practical FFR spectrum sharing scenarios, which may lead to certain performance degradation of existing algorithms in real LEO system deployments [30]. Third, existing schemes have not yet achieved effective collaborative optimization of robustness, user fairness and computational complexity, and thus may not fully meet the edge user quality of service (QoS) requirements and onboard processing capability constraints in practical applications [31].

To fill these gaps, this paper focuses on LEO satellite uplink multi-beam systems in FFR-based spectrum sharing scenarios, and proposes a low-complexity Hybrid Optimized Robust Beamforming (HORBA) algorithm. The core design objective of the algorithm is to maximize system sum rate while guaranteeing per-user minimum average signal to interference plus noise ratio, cross system interference temperature limit and maximum transmit power constraint for each user. The main contributions of this work can be summarized as follows:

1. A robust joint optimization model is constructed for FFR spectrum sharing scenarios. The model comprehensively characterizes the coupling effect of random DOA estimation errors, outdated CSI, adjacent beam interference and cross-system interference. Reasonable constraints are introduced into the model to realize the collaborative modeling of transmission robustness, user fairness and interference controllability.
2. A low-complexity solution algorithm is designed based on the BCD framework. The original non-convex problem is decoupled into two convex subproblems. The closed-form solution of beamforming vectors is derived via generalized eigenvalue decomposition and Gram–Schmidt orthogonalization. The power optimization subproblem is convexified via first-order Taylor expansion with a robust upper bound. These designs significantly reduce the computational complexity of the algorithm and adapt to the limited onboard processing capability.
3. Multi-scenario Monte Carlo simulations are carried out to verify the effectiveness of the proposed algorithm. Numerical results show that the proposed algorithm outperforms state-of-the-art benchmark schemes including the FR7 scheme, worst-case robust beamforming and sCSI-based multi-beam joint processing. The algorithm can well adapt to the practical constraints of LEO satellite IoT communication systems.

The rest of this paper is organized in the following structure. Section 2 establishes the system model and DOA estimation error model, and formulates the joint optimization problem. Section 3 elaborates the design principle and complete execution flow of the proposed HORBA algorithm. Section 4 verifies the performance of the proposed algorithm and benchmark schemes through multi-scenario numerical simulations. Finally, Section 5 concludes the whole paper and prospects relevant future research directions.

## 2. System Model and Problem Formulation

### 2.1. System Model

**Figure 1:**
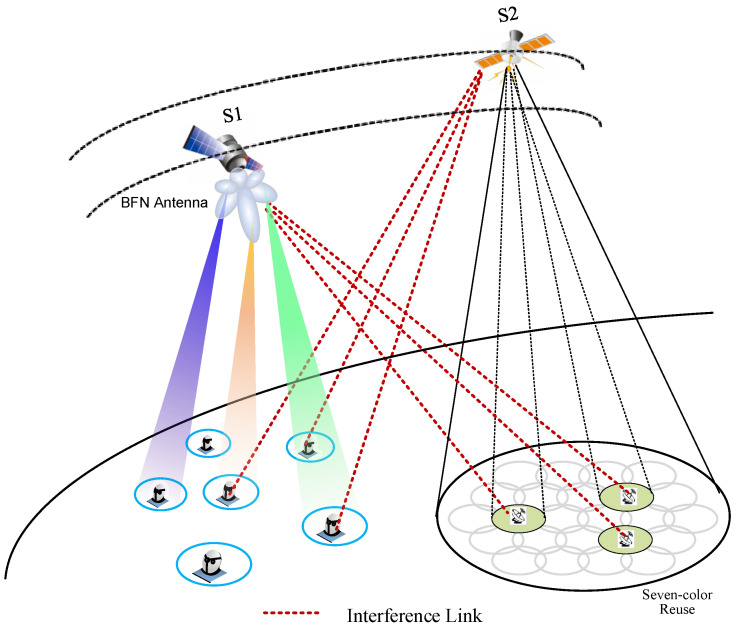
Two spectrum-sharing LEO satellites, where the red line denotes the interference link.

We consider an uplink multi-user LEO satellite communication system in a spectrum sharing scenario, where two co-frequency LEO satellite communication systems, denoted as $S_{1}$ and $S_{2}$, coexist. Among them, $S_{1}$ is the target system, equipped with a fully digital beamforming network (BFN), and adopts a uniform rectangular array (URA) with $N=M_{x}\times M_{y}$ antenna elements to serve *K* single-antenna users, where N≫K is satisfied to ensure sufficient spatial degrees of freedom for beamforming. The fully digital BFN can realize flexible adjustment of beam pointing to adapt to the dynamic communication requirements of multiple users. $S_{2}$ is the coexisting system, equipped with a fixed beamforming network, and adopts a URA with $N_{2}=M_{x2}\times M_{y2}$ antenna elements to serve *L* single-antenna users. Its beam pointing is fixed, and multi-color frequency reuse technology is adopted to suppress inter-beam interference. Users of both systems are randomly distributed within the beam coverage of $S_{1}$, the signals transmitted by all users are narrowband signals, and the transmitted signals of each user are independent of each other. The system model is illustrated in Figure 1.

The satellite-ground link of LEO satellites is a typical shadowed Rician multipath channel. Considering the key factors such as angle estimation error, Doppler shift, and propagation delay comprehensively, the received signal of the *k*-th user at the $S_{1}$ satellite can be expressed as

(1) $$y_{k}\left(t\right)=\ell_{k}\cdot g_{k}\cdot e^{j2\pi \left(t\cdot f_{d,k}-f_{0}\cdot \tau_{k}\right)}\cdot a^{H}\left(\theta_{k},\phi_{k}\right)x_{1k}\left(t\right)+n_{k}\left(t\right)$$

where $\ell_{k}$ is the free space path loss of the satellite-ground link, with the expression $\ell_{k}={\left(\frac{\lambda }{4\pi d_{k}}\right)}^{2}$, where $d_{k}$ is the propagation distance between the satellite and the user, and λ is the carrier wavelength; $g_{k}$ is the small-scale fading coefficient, which follows the Rician distribution, i.e., $g_{k}∼CN\left(\sqrt{\frac{K_{R}}{K_{R}+1}},\frac{1}{2(K_{R}+1)}\right)$, where $K_{R}$ is the Rician factor; $e^{j2\pi \left(t\cdot f_{d,k}-f_{0}\cdot \tau_{k}\right)}$ is the time-varying phase offset term caused by the high-speed motion of the LEO satellite, $f_{d,k}$ is the Doppler shift corresponding to the *k*-th user, $\tau_{k}$ is the propagation delay of the satellite-ground link, and $f_{0}$ is the carrier center frequency; and $a(\theta_{k},\phi_{k})$ is the array steering vector corresponding to the direction of arrival (elevation angle $\theta_{k}$, azimuth angle $\phi_{k}$). For a uniform rectangular array, its expression is

(2) $$\begin{array}{cc} a(\theta_{k},\phi_{k})= & \frac{1}{\sqrt{N}}{\left[1,\exp\left(j2\pi \frac{d_{e}}{\lambda }\sin\theta_{k}\cos\phi_{k}\right),\ldots ,\exp\left(j2\pi \left(M_{x}-1\right)\frac{d_{e}}{\lambda }\sin\theta_{k}\cos\phi_{k}\right)\right]}^{T}\\ \; & ⊗{\left[1,\exp\left(j2\pi \frac{d_{e}}{\lambda }\cos\theta_{k}\right),\ldots ,\exp\left(j2\pi \left(M_{y}-1\right)\frac{d_{e}}{\lambda }\cos\theta_{k}\right)\right]}^{T} \end{array}$$

where ⊗ denotes the Kronecker product; $d_{e}$ is the element spacing, which is taken as λ/2 to avoid mutual coupling interference between elements; x(t) is the user transmitted signal; and $n_{k}\left(t\right)$ is the additive white Gaussian noise (AWGN), which follows the distribution $CN\left(0,\sigma_{n}^{2}\right)$.

### 2.2. Angle Error Model

It should be noted that the 2D azimuth-elevation angle domain model adopted in this paper is a widely used classical model for LEO satellite communications. We choose this model because it can greatly reduce computational complexity while ensuring sufficient accuracy, which is well suited for onboard real-time processing. The core innovation of this paper is not to propose a new angle model but to develop a Hybrid Optimized Robust Beamforming framework that can tolerate random DOA estimation errors based on this classical model.

In LEO satellite communication scenarios, the uncertainty of angle estimation originates from the coupling effect of dynamic disturbances at the satellite end, propagation distortion of satellite-ground links, and inherent limitations of measurement equipment. Define the angle estimation errors of the *k*-th user as $\Delta \theta_{k}={\hat{\theta }}_{k}\theta_{k}$ and $\Delta \phi_{k}={\hat{\phi }}_{k}-\phi_{k}$, where ${\hat{\theta }}_{k}$ and ${\hat{\phi }}_{k}$ are the estimated angle values, and $\theta_{k}$ and $\phi_{k}$ are the true angle values. Without loss of generality, it is assumed that $\Delta \theta_{k}$ and $\Delta \phi_{k}$ follow a zero-mean normal distribution with a standard deviation of σ=aε. To accurately describe the fluctuation range of angle estimation errors, the angle uncertainty set is defined as   

(3) $$\Theta_{k}=\left\{\left(\theta_{k},\phi_{k}\right)|\theta_{k}\in \left[{\hat{\theta }}_{k}-\varepsilon ,{\hat{\theta }}_{k}+\varepsilon \right],\phi_{k}\in \left[{\hat{\phi }}_{k}-\varepsilon ,{\hat{\phi }}_{k}+\varepsilon \right]\right\},\;k=1,2,\ldots ,K$$

In complex propagation scenarios such as heavy rain and severe ionospheric scintillation, the angle estimation error will further increase. For this reason, an error enhancement coefficient $\alpha_{k}\in \left[0,1\right]$ is introduced to characterize the degree of error amplification, and the error distribution is corrected as

(4) $$\Delta \theta_{k}∼N\left(0,{\left(a\varepsilon \cdot (1+\alpha_{k})\right)}^{2}\right),\;\Delta \phi_{k}∼N\left(0,{\left(a\varepsilon \cdot (1+\alpha_{k})\right)}^{2}\right)$$

where *a* is a scaling coefficient determined by the propagation environment, which is set to 1 in this paper without loss of generality.

Angle estimation errors will directly lead to deviations in the array steering vector, thereby degrading beamforming performance. In the scenario of small angle errors, the estimated steering vector $a({\hat{\theta }}_{k},{\hat{\phi }}_{k})$ can be expanded by the first-order Taylor series around the true value $\left(\theta_{k},\phi_{k}\right)$ to approximate the deviation of the steering vector:

(5) $$a\left({\hat{\theta }}_{k},{\hat{\phi }}_{k}\right)\approx a\left(\theta_{k},\phi_{k}\right)+\frac{\partial a(\theta ,\phi )}{\partial \theta }{|}_{\left(\theta_{k},\phi_{k}\right)}\Delta \theta_{k}+\frac{\partial a(\theta ,\phi )}{\partial \phi }{|}_{\left(\theta_{k},\phi_{k}\right)}\Delta \phi_{k}$$

Based on the above approximate expression, the expectation of the steering vector deviation can be derived as

(6) $$E\left[\delta_{a}\right]=E\left[{∥a\left({\hat{\theta }}_{k},{\hat{\phi }}_{k}\right)-a\left(\theta_{k},\phi_{k}\right)∥}_{2}\right]\approx \sqrt{{∥\frac{\partial a(\theta_{k},\phi_{k})}{\partial \theta }∥}_{2}^{2}\sigma^{2}+{∥\frac{\partial a(\theta_{k},\phi_{k})}{\partial \phi }∥}_{2}^{2}\sigma^{2}}$$

It can be seen from (6) that the expectation of the steering vector deviation is positively correlated with the standard deviation σ of the angle error, which verifies the necessity of robust beamforming design.

### 2.3. Signal Model and Problem Formulation

In the considered scenario, the transmit power of the *k*-th user in $S_{1}$ is $P_{1k}$, and the total transmit power satisfies $\sum_{k=1}^{K}P_{1k}\leq P_{\max}$. The receive beam vector of satellite $S_{1}$ corresponding to the *k*-th user is $w_{k}\in C^{N\times 1}$, with the normalization constraint ${∥w_{k}∥}_{2}=1$. The additive white Gaussian noise at the satellite ends are $n_{1}∼\mathrm{CN}\left(0,\sigma_{n1}^{2}I\right)$ (satellite $S_{1}$) and $n_{2}∼\mathrm{CN}\left(0,\sigma_{n2}^{2}I\right)$ (satellite $S_{2}$), respectively.

Define the complex channel vector $h_{1k}=\ell_{k}g_{k}e^{j2\pi (tf_{d,k}-f_{0}\tau_{k})}a\left(\theta_{k},\phi_{k}\right)\in C^{N\times 1}$, which integrates path loss, small-scale fading, time-varying phase offset and array steering vector. $h_{12,l}\in C^{N\times 1}$ denotes the cross-system interference channel from the *l*-th user of $S_{2}$ to $S_{1}$. The received signal at satellite $S_{1}$ can be expressed as

(7) $$y_{1}=\sum_{k=1}^{K}\sqrt{P_{1k}}h_{1k}s_{1k}+\sum_{l=1}^{L}\sqrt{P_{2l}}h_{12,l}s_{2l}+n_{1}$$

Satellite $S_{1}$ recovers the signal of the *k*-th user through the receive beam vector $w_{k}$, i.e., ${\hat{s}}_{1k}=w_{k}^{H}y_{1}$. The corresponding SINR is

(8) $$SINR_{1k}=\frac{P_{1k}{∥w_{k}^{H}h_{1k}∥}_{2}^{2}}{\sum_{i\neq k}^{K}P_{1i}{∥w_{k}^{H}h_{1i}∥}_{2}^{2}+\sum_{l=1}^{L}P_{2l}{∥w_{k}^{H}h_{1l}∥}_{2}^{2}+\sigma_{n1}^{2}}$$

Considering the time-varying nature of LEO satellite channels and the randomness of angle errors, the average SINR (ASINR) is introduced to characterize the long-term communication performance:   

(9) $$ASINR_{1k}=\frac{P_{1k}\cdot E\left[{∥w_{k}^{H}h_{1k}∥}_{2}^{2}\right]}{E\left[\sum_{i\neq k}^{K}P_{1i}{∥w_{k}^{H}h_{1i}∥}_{2}^{2}+\sum_{l=1}^{L}P_{2l}{∥w_{k}^{H}h_{1l}∥}_{2}^{2}\right]+\sigma_{n1}^{2}}$$

Correspondingly, the system sum rate is expressed as

(10) $$R_{\mathrm{sum}}=\sum_{k=1}^{K}\log_{2}\left(1+{\mathrm{ASINR}}_{1k}\right)$$

The core optimization objective of this paper is to maximize the system sum rate of $S_{1}$, while satisfying the constraints of beam normalization, total transmit power, user minimum power, and cross-system interference temperature. The constructed optimization problem is

(11) $$\left\{\begin{array}{c} \max_{W,P}\sum_{k=1}^{K}\log_{2}\left(1+{\mathrm{ASINR}}_{1k}\right)\\ s.t.\;{∥w_{k}∥}_{2}=1,\;k=1,2,\ldots ,K\\ \;\sum_{k=1}^{K}P_{1k}\leq P_{\max}\\ \;P_{1k}\geq \gamma_{\mathrm{th}},\;k=1,2,\ldots ,K\\ \;\sum_{k=1}^{K}P_{1k}{∥w_{k}^{H}h_{S_{2}}∥}_{2}^{2}\leq I_{\mathrm{th}} \end{array}\right.$$

where $W=\left[w_{1},w_{2},\ldots ,w_{K}\right]\in C^{N\times K}$ is the beamforming matrix, $P={\left[P_{11},P_{12},\ldots ,P_{1K}\right]}^{T}\in R_{+}^{K\times 1}$ is the user transmit power vector, $\gamma_{\mathrm{th}}$ is the minimum transmit power threshold for each user, and $I_{\mathrm{th}}$ is the maximum tolerable interference power threshold for the $S_{2}$ system.

In the formulated optimization problem, the beamforming matrix W and power allocation vector P are mutually coupled, and the objective function is non-convex, making this a typical intractable non-convex optimization problem. A direct solution to this problem incurs prohibitive computational complexity, which cannot meet the stringent onboard processing constraints of LEO satellites. To address this challenge, this paper develops an efficient block coordinate descent (BCD)-based cross-iteration decoupling strategy to solve the problem.

## 3. Hybrid Optimized Robust Beamforming Algorithm Design

The core of the proposed Hybrid Optimized Robust Beamforming (HORBA) algorithm is to achieve the joint optimization of robust beamforming and power allocation in DOA estimation error scenarios, via a three-stage technical framework consisting of adaptive dynamic channel modeling, cross-iteration decoupling, and convex closed-form solution. The detailed design of the algorithm is elaborated as follows.

### 3.1. Adaptive Dynamic Channel Modeling

For LEO satellite massive MIMO systems, the time–frequency synchronization and spatial beamforming are generally designed as independent modules. The receiver can compensate for the Doppler shifts and propagation delays through mature synchronization schemes. After compensation, the equivalent baseband channel shows quasi-static characteristics within the channel coherence time. Thus, the average channel matrix is constructed based on the spatial statistics of DOA estimation errors, which is consistent with the common modeling method in robust beamforming for LEO satellite communications [13,32]. To accurately characterize the statistical characteristics of DOA estimation errors and avoid the over-conservatism of traditional worst-case robust algorithms, an adaptive dynamic sample mechanism based on angle error magnitude is proposed. Specifically, the number of channel samples is adaptively adjusted according to the standard deviation of angle error $\sigma_{\theta }$   

(12) $$N_{s}=\left\lfloor N_{0}+k\cdot \sigma_{\theta }^{2}\right\rfloor$$

where $N_{0}$ is the baseline sample number, and *k* is the proportional coefficient. In this paper, we empirically set $N_{0}=30$ and k=10 to balance modeling accuracy and computational complexity. This continuous adaptive sampling strategy achieves an optimal trade-off between statistical modeling accuracy and computational complexity, and guarantees the convergence of channel mean estimation under the shadowed Rician channel with $K_{R}=3$. Based on the angle error samples, $N_{s}$ channel realization matrices are generated, and the average channel matrix integrating the statistical characteristics of angle errors is calculated as

(13) $$H_{\mathrm{avg}}=\frac{1}{N_{s}}\sum_{i=1}^{N_{s}}H_{\mathrm{sam},i}$$

where $H_{\mathrm{sam},i}$ is the *i*-th channel realization matrix generated based on the angle error samples. This average channel matrix fully incorporates the random fluctuation characteristics of DOA estimation errors, which is the core basis for the robust beamforming design of the proposed algorithm.

### 3.2. BCD-Based Cross-Iteration Decoupling Solution

Based on the BCD framework, the original non-convex optimization problem (11) is decoupled into two independent convex subproblems: the beamforming optimization subproblem with fixed power, and the power allocation optimization subproblem with fixed beamforming. The global optimal solution of the original problem is approximated by alternately solving the two convex subproblems.

#### 3.2.1. Beamforming Optimization with Fixed Power

With the power allocation vector P fixed, the original problem is transformed into a beamforming optimization problem. Based on the average channel matrix $H_{\mathrm{avg}}$, the signal matrix and interference-plus-noise matrix of the *k*-th user are constructed as

(14) $$A_{k}=P_{1k}H_{\mathrm{avg},k}H_{\mathrm{avg},k}^{H}$$

(15) $$B_{k}=\sigma_{n1}^{2}I+\sum_{j\neq k}^{K}P_{1j}H_{\mathrm{avg},j}H_{\mathrm{avg},j}^{H}+\sum_{l=1}^{L}P_{2l}H_{\mathrm{avg},l}H_{\mathrm{avg},l}^{H}$$

where $H_{\mathrm{avg},k}$ is the average channel vector of the *k*-th user. Based on the above matrices, the SINR of the *k*-th user can be simplified as a generalized Rayleigh quotient:

(16) $$SINR_{1k}=\frac{w_{k}^{H}A_{k}w_{k}}{w_{k}^{H}B_{k}w_{k}}$$

According to matrix theory, the optimal beam vector $w_{k}$ that maximizes the above generalized Rayleigh quotient is the eigenvector corresponding to the maximum generalized eigenvalue of the matrix pair $\left(A_{k},B_{k}\right)$, which can be efficiently solved via generalized eigenvalue decomposition (GED).

To further suppress inter-user interference caused by beam overlap under angle estimation errors, Gram–Schmidt orthogonalization is performed on the solved initial beam vectors to eliminate the correlation between beam vectors of different users

(17) $$\left\{\begin{array}{c} w_{1}=\frac{v_{k1}}{{∥v_{k1}∥}_{2}}\\ w_{k}=\frac{v_{k1}-\sum_{i=1}^{k-1}\left(w_{i}^{H}v_{k1}\right)w_{i}}{{∥v_{k1}-\sum_{i=1}^{k-1}\left(w_{i}^{H}v_{k1}\right)w_{i}∥}_{2}},\;k=2,\ldots ,K \end{array}\right.$$

where $v_{k1}$ is the initial beam vector obtained via GED. With the fixed power allocation, the optimal beam vector $v_{k1}$ can be obtained by maximizing the generalized Rayleigh quotient, which is solved by generalized eigenvalue decomposition. It should be clarified that Gram–Schmidt orthogonalization is utilized as an auxiliary operation to suppress inter-user interference in each BCD iteration. The optimal beam vector will be recalculated by GED in the next iteration, so the overall optimality of the algorithm will not be damaged. For the massive MIMO scenario with more antennas than users, the performance loss caused by orthogonalization is quite small due to the asymptotic orthogonality of channels.

#### 3.2.2. Power Allocation Optimization with Fixed Beamforming

With the optimized beamforming matrix W fixed, the original problem is transformed into a power allocation optimization problem. To simplify the model, four auxiliary variables are introduced:

(18) $$\left\{\begin{array}{c} a_{k}={∥w_{k}^{H}h_{1k}∥}_{2}^{2}\\ b_{ki}={∥w_{k}^{H}h_{1i}∥}_{2}^{2}\\ c_{k}=\sum_{l=1}^{L}P_{2l}{∥w_{k}^{H}h_{1l}∥}_{2}^{2}+\sigma_{n1}^{2}\\ d_{k}={∥w_{k}^{H}h_{S_{2}}∥}_{2}^{2} \end{array}\right.$$

Based on the above auxiliary variables, the power allocation optimization problem can be transformed into

(19) $$\left\{\begin{array}{c} \max_{P}\sum_{k=1}^{K}\log_{2}\left(1+\frac{a_{k}P_{1k}}{\sum_{i\neq k}^{K}b_{ki}P_{1i}+c_{k}}\right)\\ s.t.\;\sum_{k=1}^{K}P_{1k}\leq P_{\max}\\ \;P_{1k}\geq \gamma_{\mathrm{th}},\;k=1,2,\ldots ,K\\ \;\sum_{k=1}^{K}d_{k}P_{1k}\leq I_{\mathrm{th}} \end{array}\right.$$

This problem is a standard convex optimization problem, which can be efficiently solved by the interior-point method. By alternately iteratively solving the above two convex subproblems until the convergence condition is met, the optimal beamforming matrix and power allocation vector of the original problem can be obtained.

The complete execution flow of the proposed HORBA algorithm is summarized in Algorithm 1, and the computational complexity of each core module is decomposed and analyzed in Table 1.

As shown in Table 1, the overall complexity of the proposed algorithm is dominated by the generalized eigenvalue decomposition (GED) module with a magnitude of $O(K\cdot N^{3})$, which is several orders of magnitude lower than the $O(N^{6})$ complexity of traditional SDP/SOCP-based robust algorithms. This low theoretical complexity provides a feasible basis for implementation on resource-constrained onboard processors.
**Algorithm 1** HORBA algorithm execution flow.**Require:** Antenna array size *N*, number of users *K*, angle error threshold ε, maximum total transmit power $P_{\max}$, single-user minimum power threshold $\gamma_{\mathrm{th}}$, cross-system interference threshold $I_{\mathrm{th}}$, convergence threshold $\varepsilon_{\mathrm{conv}}$, maximum iterations $T_{\max}$ **Ensure:** Optimal beamforming matrix $W^{*}$, optimal power allocation vector $P^{*}$
  1:Randomly generate initial beam matrix $W^{\left(0\right)}$ satisfying ${∥w_{k}^{\left(0\right)}∥}_{2}=1$  2:Initialize power vector $P^{\left(0\right)}=\gamma_{\mathrm{th}}+\left(P_{\max}-K\cdot \gamma_{\mathrm{th}}\right)/K$  3:Set iteration counter t=0  4:**while** $t<T_{\max}$ **do**  5:   **Adaptive Channel Sampling**:  6:      Adjust sample number $N_{s}$ by (12), generate $N_{s}$ channel realization matrices, and compute average channel matrix $H_{\mathrm{avg}}$ by (13)  7:   **Beamforming Optimization**:  8:      For each user k=1,2,…,K, construct signal matrix $A_{k}$ and interference-plus-noise matrix $B_{k}$ by (14) and (15)  9:      Solve initial beam vector $v_{k1}$ via generalized eigenvalue decomposition (GED) of matrix pair $\left(A_{k},B_{k}\right)$10:      Perform Gram–Schmidt orthogonalization by (17) to obtain optimized beam matrix $W^{\left(t+1\right)}$11:   **Power Allocation Optimization**:12:      Calculate auxiliary variables by (18)13:      Solve convex power optimization problem (19) via interior-point method to obtain optimal power vector $P^{\left(t+1\right)}$14:   **Convergence Judgment**:15:      Compute iteration error $\Delta ={∥W^{\left(t+1\right)}-W^{\left(t\right)}∥}_{F}+{∥P^{\left(t+1\right)}-P^{\left(t\right)}∥}_{2}$16: 17:   **if** $\Delta <\varepsilon_{\mathrm{conv}}$ **then**18:           **break**19: 20:   **else**21:           Set t=t+122: 23:   **end if**24:**end while**


In terms of practical execution efficiency, simulation results show that the algorithm can typically achieve more than 90% of its maximum performance within 10 iterations for the practical DOA estimation error scenarios considered in this work. After the initial beam alignment phase, only 3–5 iterations are generally sufficient to maintain stable performance during continuous beam tracking, which helps to reduce the average computational load during long-term system operation. All simulation parameters are consistent with the typical characteristics of current commercial LEO satellite communication systems, which helps to ensure the conclusions have certain practical engineering reference value.

Convergence Analysis: The proposed HORBA algorithm is based on the block coordinate descent (BCD) framework, which alternately optimizes the beamforming matrix W and the power allocation vector P. In each iteration, the objective function is maximized with respect to one block variable while holding the other block variables constant. Since the objective function is continuous and differentiable with respect to each block variable, and each subproblem has a unique optimal solution, the sequence of objective function values generated by the algorithm is strictly non-decreasing and upper bounded. According to the monotone convergence theorem, the proposed algorithm is guaranteed to converge to a stationary point of the original non-convex problem.

## 4. Numerical Results and Analysis

In this section, we provide simulation results to evaluate the performance of the proposed HORBA algorithm. The main simulation parameters are listed in Table 2. The IB, S-NAB, CWRB and 7-FRB are used as the benchmarking schemes. The IB scheme can be viewed as the ideal scheme, S-NAB exploits the nominal angle CSI without robust compensation, CWRB exploits the worst-case CSI defined in the existing robust beamforming works, and 7-FRB adopts the fixed 7-color frequency reuse scheme for practical satellite systems.

**Figure 2:**
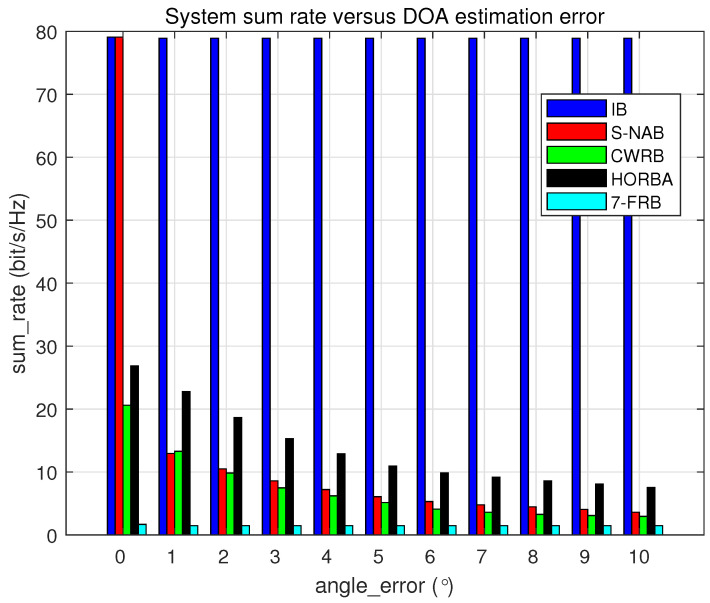
System sum rate versus DOA estimation error.

Figure 2 shows the system sum rate of various algorithms under different DOA estimation errors. It can be seen that the sum rate of the IB algorithm is not affected by angle errors, serving as the theoretical upper bound. In the 0° error-free scenario, the sum rate of the S-NAB algorithm is close to that of the IB algorithm, but it drops sharply with the increase in angle errors. The CWRB algorithm alleviates the performance loss to a certain extent, but its over-conservative design leads to a significantly lower sum rate than the proposed HORBA algorithm in the full error range. The 7-FRB algorithm maintains a consistently low sum rate due to its fixed beam design. In contrast, the proposed HORBA algorithm exhibits significant performance advantages in all error scenarios, which verifies the effectiveness of its robust design.

**Figure 3:**
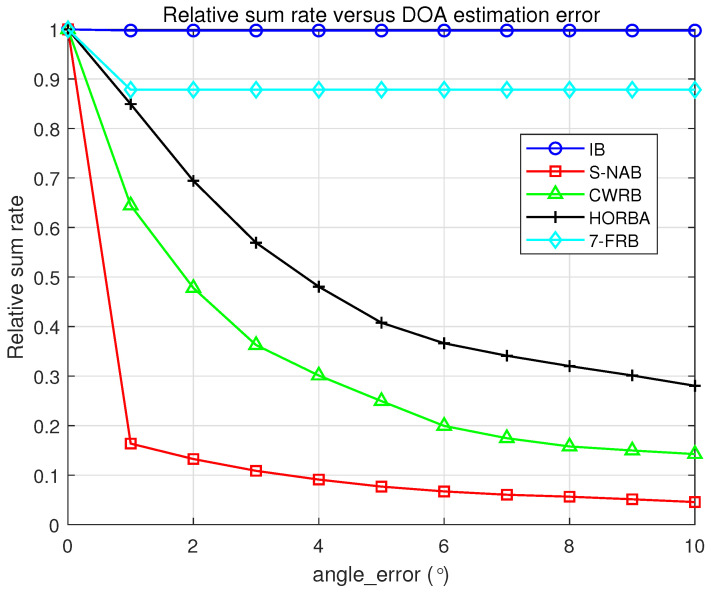
Relative sum rate versus DOA estimation error.

Figure 3 shows the relative sum rate (normalized by the sum rate at 0° error) of different algorithms, which directly reflects the robustness of the algorithms. It can be seen that the proposed HORBA algorithm achieves the best robustness performance among all practical algorithms, with a relative sum rate of 0.48 at 4° error, which is 5.33 times that of the S-NAB algorithm and 1.6 times that of the CWRB algorithm. This result validates that the proposed algorithm can effectively maintain stable system performance under DOA estimation error scenarios.

**Figure 4:**
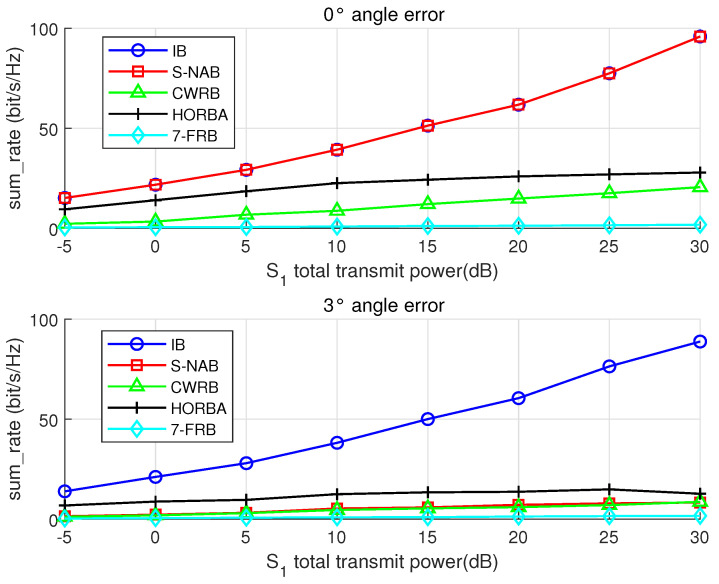
System sum rate versus total transmit power.

Figure 4 shows the relationship between the total transmit power and the system sum rate under 0° and 3° angle error scenarios. In the 0° error-free scenario, the sum rates of the IB and S-NAB algorithms increase linearly with the increase in transmit power, and their performance is almost identical. The sum rate of the proposed HORBA algorithm increases steadily and is always significantly higher than that of the CWRB and 7-FRB algorithms. In the 3° angle error scenario, the performance of the S-NAB algorithm is severely degraded, and the increase in transmit power cannot be effectively converted into system capacity gain. In contrast, the proposed HORBA algorithm still maintains good power utilization efficiency. At a transmission power of 15 dBm, the total rate of the proposed algorithm decreases by 44.76% in performance, while S-NAB decreases by 88.53% and the CWRB algorithm decreases by 55.35%.

**Figure 5:**
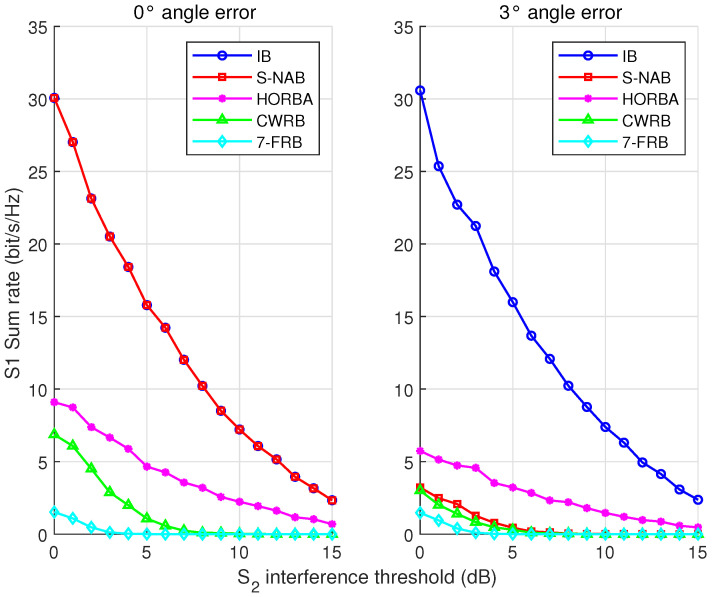
System sum rate versus $S_{2}$ SINR threshold.

Figure 5 shows the relationship between the minimum SINR threshold of the $S_{2}$ system and the sum rate of the $S_{1}$ system. It can be seen that with the increase in the SINR threshold of the $S_{2}$ system, the sum rate of all algorithms shows a downward trend. In the 3° angle error scenario, the S-NAB algorithm has the most severe performance degradation due to the aggravated interference leakage caused by angle errors. In contrast, the proposed HORBA algorithm can suppress the interference leakage caused by angle errors through robust beamforming design; under the conditions of 3° error and 10 dB SINR threshold of the $S_{2}$ system, it can still maintain 78.5% of the sum rate in the 0° scenario, far better than the benchmark algorithms.

**Figure 6:**
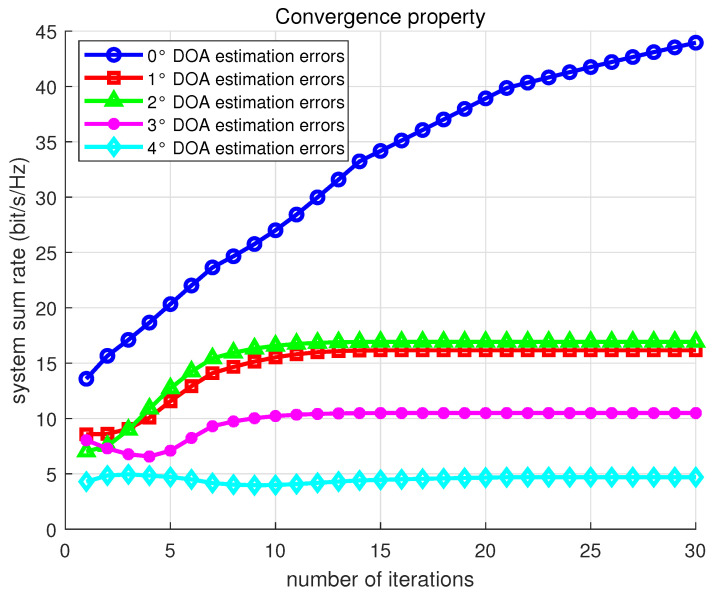
Convergence verification of the HORBA algorithm under different DOA estimation errors.

To verify the convergence property of the proposed HORBA algorithm, Figure 6 depicts the system sum rate versus the number of iterations under different DOA estimation errors, i.e., 0°, 1°, 2°, 3°, and 4°. As illustrated, the sum rate increases monotonically with the number of iterations. The ideal 0° error-free scenario converges to its maximum value within approximately 20 iterations, while all practical DOA estimation error scenarios (1°–4°) reach over 90% of their peak performance within 10 iterations. As the DOA estimation error increases, the final achievable sum rate decreases gradually, which is mainly attributed to the aggravated inter-user interference and beam pointing mismatch. The observed convergence trend is consistent with the theoretical analysis in Section 3, which supports the effectiveness of the BCD-based iterative optimization framework. Even under the maximum 4° DOA estimation error considered in this work, the algorithm remains convergent, indicating its good robustness against angle estimation inaccuracies.

**Figure 7:**
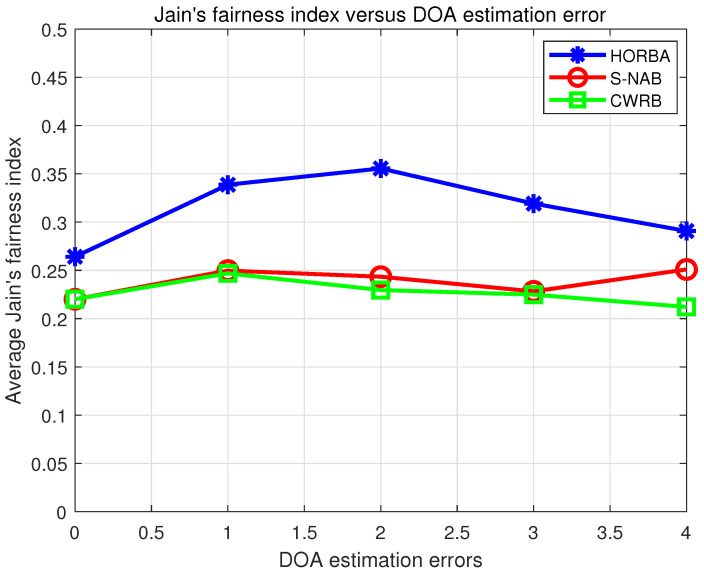
Average Jain’s fairness index versus DOA estimation error.

To evaluate the user fairness of different algorithms, Jain’s fairness index and the cumulative distribution function (CDF) of user rates are adopted. Figure 7 shows the average Jain’s fairness index versus DOA estimation error for the proposed HORBA algorithm and two benchmark algorithms, i.e., S-NAB and CWRB. It is clear that the proposed HORBA algorithm achieves the highest fairness index under all DOA estimation error conditions, which indicates that the rate distribution among users is more balanced. In contrast, the non-robust S-NAB and traditional robust CWRB algorithms suffer from severe user rate polarization, resulting in poor fairness performance.

**Figure 8:**
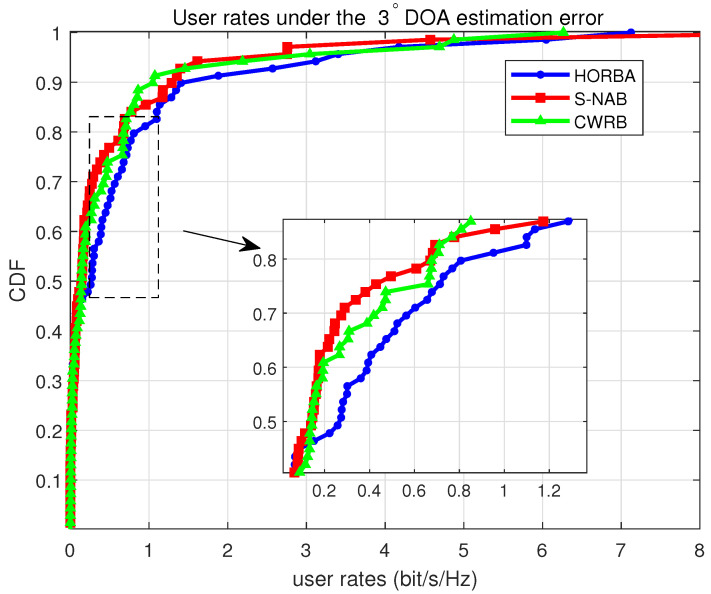
CDF of user rates under $3^{\circ }$ DOA estimation error.

Figure 8 depicts the cumulative distribution function (CDF) of user rates under the typical 3° DOA estimation error. In the low-rate region, the CDF curve of the conventional robust CWRB rises the fastest, indicating that a large proportion of users suffer from severe rate degradation, followed by the non-robust S-NAB. The proposed HORBA algorithm exhibits the slowest rise in this region, demonstrating its effectiveness in protecting edge users from extremely low data rates. As the rate increases, the three curves gradually approach each other due to the fixed total transmit power constraint, which limits the upper bound of user rates. Despite the visual proximity in the mid-to-high rate region, HORBA achieves the most balanced rate distribution without obvious rate polarization, thus verifying its superior fairness performance.

## 5. Conclusions

In this paper, we have investigated the uplink multi-beam transmission in a spectrum-sharing LEO satellite communication system. To ensure the quality of service for each user, we have designed a HORBA scheme to maximize the system sum rate while guaranteeing the minimum average SINR of all users. Then, by using the adaptive dynamic channel modeling, block coordinate descent framework and generalized eigenvalue decomposition, we proposed an iterative algorithm to solve the beamforming optimization problem with the imperfect DOA estimation in the satellite-ground links. Finally, numerical results revealed that our proposed algorithm outperforms the existing works and can reduce the effect of the DOA estimation error and the cross-system interference, thereby improving the system performance and robustness.

## Figures and Tables

**Table 1:** Algorithm complexity decomposition.

Core Module	Complexity Expression	Core Characteristic
Adaptive Channel Modeling	$O\left(T\cdot N_{s}\cdot N\cdot K\right)$	Controllable, fixed $N_{s}$
GED Beam Solving	$O\left(T\cdot K\cdot N^{3}\right)$	Core complexity term
Beam Orthogonalization	$O\left(T\cdot K^{2}\cdot N\right)$	Far lower than GED (N≫K)
Power Optimization	$O\left(T\cdot T_{1}\cdot K^{2}\right)$	Negligible (K≪N)
**Overall Complexity**	$O\left(T\cdot \left(N_{s}\cdot N\cdot K+K\cdot N^{3}+K^{2}\cdot N+T_{1}\cdot K^{2}\right)\right)$	

Note: Bold text indicates the dominant computational complexity term.

**Table 2:** Core simulation parameter settings.

Parameter Name	Parameter Value
Carrier Center Frequency	14.5 GHz (Ku band)
Antenna Array Size of $S_{1}$	16×16 (256 elements)
Orbit Inclination	$S_{1}$: $53.8^{\circ }$; $S_{2}$: $87.9^{\circ }$
Orbit Altitude	$S_{1}$: 1100 km; $S_{2}$: 1200 km
Number of Users of $S_{1}$ (*K*)	14
Channel Rician Factor ($K_{R}$)	3
Maximum Total Transmit Power ($P_{\max}$)	25 dBm
Single-User Minimum Power Threshold ($\gamma_{\mathrm{th}}$)	$0.1\times P_{\max}/K$
Minimum SINR Threshold of $S_{2}$	0 dB
System Bandwidth	250 MHz
Noise Temperature	300 K
Standard Deviation of Angle Error (ε)	0°∼ 10°
Convergence Threshold ($\varepsilon_{\mathrm{conv}}$)	$10^{-4}$
Baseline Sample Number ($N_{0}$)	30
Adaptive Sampling Proportional Coefficient (*k*)	10

## Data Availability

The original contributions presented in this study are included in the article. Further inquiries can be directed to the corresponding author.
